# Mass balance and metabolite profiling of ^14^C-guadecitabine in patients with advanced cancer

**DOI:** 10.1007/s10637-019-00854-9

**Published:** 2019-10-11

**Authors:** Jeroen Roosendaal, Hilde Rosing, Luc Lucas, Abadi Gebretensae, Alwin D. R. Huitema, Marloes G. van Dongen, Jos H. Beijnen, Aram Oganesian

**Affiliations:** 1 Department of Pharmacy & Pharmacology, Netherlands Cancer Institute – Antoni van Leeuwenhoek, Amsterdam, The Netherlands; 2 Division of Pharmacoepidemiology and Clinical Pharmacology, Science Faculty, Utrecht Institute for Pharmaceutical Sciences, Utrecht University, Utrecht, The Netherlands; 3 Department of Clinical Pharmacy, University Medical Center Utrecht, Utrecht University, Utrecht, The Netherlands; 4 Division of Medical Oncology, Netherlands Cancer Institute – Antoni van Leeuwenhoek, Amsterdam, The Netherlands; 5 Division of Pharmacology, Netherlands Cancer Institute – Antoni van Leeuwenhoek, Amsterdam, The Netherlands; 6 Astex Pharmaceuticals, Inc., Pleasanton, California, USA

**Keywords:** Guadecitabine, ß-decitabine, Pharmacokinetics, Mass balance, Metabolite, ^14^C-radiolabeled

## Abstract

*Purpose* The objective of this mass balance trial was to determine the excretory pathways and metabolic profile of the novel anticancer agent guadecitabine in humans after administration of a ^14^C-radiolabeled dose of guadecitabine. *Experimental design* Included patients received at least one cycle of 45 mg/m^2^ guadecitabine subcutaneously as once-daily doses on Days 1 to 5 of a 28-day cycle, of which the 5th (last) dose in the first cycle was spiked with ^14^C-radiolabeled guadecitabine. Using different mass spectrometric techniques in combination with off-line liquid scintillation counting, the exposure and excretion of ^14^C-guadecitabine and metabolites in the systemic circulation, excreta, and intracellular target site were established. *Results* Five patients were enrolled in the mass balance trial. ^14^C-guadecitabine radioactivity was rapidly and almost exclusively excreted in urine, with an average amount of radioactivity recovered of 90.2%. After uptake in the systemic circulation, guadecitabine was converted into ß-decitabine (active anomer), and from ß-decitabine into the presumably inactive metabolites M1-M5. All identified metabolites in plasma and urine were ß-decitabine related products, suggesting almost complete conversion via cleavage of the phosphodiester bond between ß-decitabine and deoxyguanosine prior to further elimination. ß-decitabine enters the intracellular activation pathway, leading to detectable ß-decitabine-triphosphate and DNA incorporated ß-decitabine levels in peripheral blood mononuclear cells, providing confirmation that the drug reaches its DNA target site. *Conclusion* The metabolic and excretory pathways of guadecitabine and its metabolites were successfully characterized after subcutaneous guadecitabine administration in cancer patients. These data support the clinical evaluation of safety and efficacy of the subcutaneous guadecitabine drug product.

**Electronic supplementary material:**

The online version of this article (10.1007/s10637-019-00854-9) contains supplementary material, which is available to authorized users.

## Introduction

**Fig. 1:**
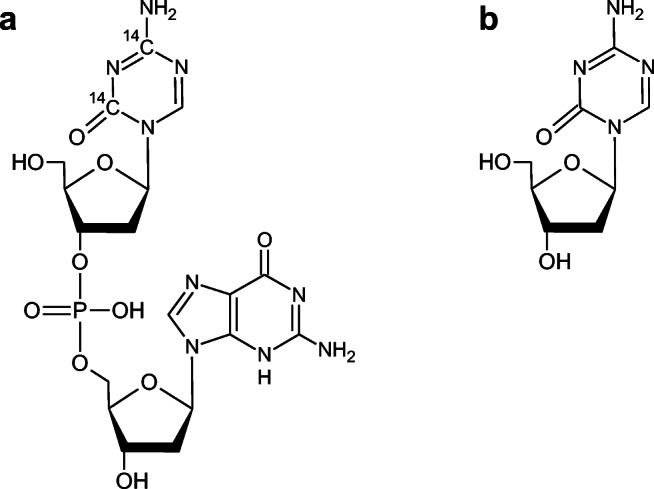
Molecular structures of (**a**) ^14^C-guadecitabine (position of ^14^C-radiolabels marked) and (**b**) its active metabolite ß-decitabine

Guadecitabine is a dinucleotide of the Food and Drug Administration (FDA)- and European Medicines Agency (EMA)-approved drug ß-decitabine (Dacogen®) and endogenous deoxyguanosine linked by a phosphodiester bond (Fig. 1). In vivo cleavage of the phosphodiester bond of guadecitabine in the systemic circulation results in a gradual release of ß-decitabine, and, as a result, in prolonged systemic exposure as compared to intravenous ß-decitabine administration. ß-decitabine is an epigenetic hypomethylating agent that requires intracellular activation and DNA incorporation before it can exert its hypomethylating effect. [1] Since ß-decitabine activity is S-phase dependent, more prolonged exposure should result in more S-phase cancer cells exposed to the action of ß-decitabine, which might result in better treatment efficacy.

Guadecitabine is currently being investigated in phase II/III clinical trials in patients with several types of solid tumors and hematological malignancies. Pharmacokinetic trials display a prolonged systemic exposure to ß-decitabine after subcutaneous guadecitabine administration, compared to ß-decitabine exposure after intravenous infusion of ß-decitabine. [2, 3] However, a complete overview of the metabolic fate and excretory pathways after subcutaneous administration of guadecitabine in humans is lacking. Information on the metabolic fate of a drug candidate is ideally investigated using a ^14^C-radiolabeled absorption, distribution, metabolism and excretion (ADME) trial, also called a mass balance trial. [4–6] Data on the metabolism and excretion of ß-decitabine after intravenous infusion of ß-decitabine are available, but results from a mass balance trial were never published in detail. [7]

The need to perform a mass balance trial in clinical anticancer drug development is emphasized by both the FDA and the EMA. [8–10] The investigation of guadecitabine in a mass balance trial will provide an estimate of basic pharmacokinetic parameters, an assessment of the routes and rates of elimination of radioactivity, and identification of metabolites and metabolic pathways. By obtaining results on these important drug characteristics, the mass balance trial aids to an appropriate understanding of the safety and efficacy of subcutaneous guadecitabine. Here, the results on the clinical mass balance trial of guadecitabine after subcutaneous administration of ^14^C-radiolabeled guadecitabine to cancer patients are described.

## Materials and methods

### Study design

This was a Phase 1, single-center, open-label, non-controlled, mass balance and metabolite profiling study (EudraCT 2015–003083-36) with ^14^C-radiolabeled guadecitabine in subjects with hematological or solid tumors who qualified for investigational treatment with guadecitabine following the inclusion and exclusion criteria. Subjects received at least one cycle of 45 mg/m^2^ guadecitabine as once-daily subcutaneously administered doses on Days 1 to 5 of a 28-day cycle, of which the 5th (last) dose in the first cycle was spiked with approximately 46.25 kBq/mg (1.25 μCi/mg) of ^14^C-radiolabeled guadecitabine. Additional cycles were with unlabeled guadecitabine.

### Patients

Subjects ≥18 years diagnosed with acute myeloid leukemia (AML), myelodysplastic syndrome (MDS) or solid tumors that qualified for treatment with guadecitabine were eligible for inclusion. Subjects needed to have an Eastern Cooperative Oncology Group (ECOG) performance status of 0–2, and acceptable organ function, as evidenced by laboratory data: aspartate aminotransferase (AST) and alanine aminotransferase (ALT) <3x the upper limit of normal (ULN), total serum bilirubin ≤35 μmol/L, serum creatinine levels ≤1.25x ULN or calculated by Cockcroft-Gault formula ≥50 mL/min, and an internationalized normalized ratio (INR) ≤2.3. Subjects who received radiation therapy, other locoregional therapy, or chemotherapy within 4 weeks prior to the first dose of study drug, or who previously participated in human mass balance studies and treated with radiolabeled drug were excluded.

### Study medication

Guadecitabine was provided by Astex Pharmaceuticals, Inc. and administered as a subcutaneous injection at a concentration of 100 mg/mL in the custom diluent comprising of propylene glycol/glycerin/ethanol vehicle (65:25:10, *v*/v/v/). Radiolabeled ^14^C-guadecitabine was manufactured, packed and labeled by Quotient Clinical (Ruddington, Nottingham, UK). ^14^C-guadecitabine was labeled with up to two ^14^C-labels in the nucleobase part of the ß-decitabine part of the guadecitabine molecule (Fig. 1). The composition and strength of the radiolabeled formulation were the same as for unlabeled guadecitabine, with the exception that ^14^C-radiolabeled guadecitabine was mixed with unlabeled guadecitabine at a ratio of approximately 1:250 to achieve a median specific activity of 0.94 μCi/mg guadecitabine in the final drug product intended for subcutaneous administration.

### Chemicals

ß-Decitabine was purchased from Alsachim (Illkirch, France). Guadecitabine and decitabine [^13^C_2_, ^15^N_4_] were provided by Astex Pharmaceuticals, Inc. and manufactured by Clauson-Kaas (Farum, Denmark) and Asclep Pharmard (Newark, DE, USA), respectively. Acetonitrile, methanol, and water (all Supra-Gradient grade) were supplied by Biosolve Ltd. (Valkenswaard, The Netherlands). Formic acid (≥98%; analytical grade), 2-propanol (>99.8%), hydrogen peroxide (30%, *w*/w), ethylenediaminetetraacetic acid (EDTA) 99% and sodium hydroxide (50%, w/w) were provided by Merck (Amsterdam, The Netherlands). Ammonium formate was purchased from Sigma Aldrich (Zwijndrecht, The Netherlands). Ultima Gold™ and Solvable™ were obtained from PerkinElmer (Groningen, The Netherlands). Water was purchased from B. Braun (Oss, The Netherlands).

### Sample collection and processing

#### Whole blood, plasma, and plasma-ultrafiltrate

Blood sampling for plasma and whole blood analysis was performed at the following time points: pre-dose, 0.5, 0.75, 1, 1.5, 2, 2.5, 4, 6, 8 h on Day 1 and at pre-dose, 0.5, 0.75, 1, 1.5, 2, 2.5, 4, 6, 8, 12 and 24 h on Day 5. From Day 6 onwards, blood was collected once daily until hospital release. For each time point, 10 mL of blood was drawn via a peripheral line using potassium ethylenediaminetetra-acetic acid (K_2_EDTA) tubes (Vacutainer®, Becton, Dickinson and Company, Franklin Lakes, NJ, USA). Prior to sample collection, all K_2_EDTA tubes were spiked with 10 mg/mL tetrahydrouridine (THU) to obtain a final concentration of 0.1 mg/mL directly after collection to prevent cytidine deaminase mediated degradation of ß-decitabine. To obtain plasma, 6 mL of whole blood was centrifuged (2000 g, 10 min, 4 °C). The remaining 4 mL of whole blood was directly stored without any further pretreatment. At the following time points, an additional 4 mL of blood was collected for plasma-ultrafiltrate radioactivity analysis: pre-dose, 2, 4, 8, and 24 h post-^14^C-guadecitabine administration. Plasma-ultrafiltrate was prepared by centrifuging 1 mL of plasma using Millipore Centrifree YM-30 filters (1000 g, 30 min, 4 °C). Separate aliquots were made for whole blood, plasma, and plasma-ultrafiltrate radioactivity measurements to be analyzed directly after processing. Aliquots for liquid chromatography coupled to tandem mass spectrometry (LC-MS/MS) analysis and metabolite profiling experiments were prepared from whole blood and plasma only, and stored at −80 °C prior to analysis.

#### Urine and feces

Urine was collected each time the bladder was emptied for the first 24 h after administration, and in 24 h intervals from Day 2 onwards. Urine was stored in polyethylene containers at 2–8 °C for the duration of the collection period. After each collection period, urine samples were mixed thoroughly, weighed, portioned into aliquots, and frozen at −80 °C. Separate aliquots were made in polypropylene tubes for LC-MS/MS and liquid scintillation counting (LSC) analysis.

Feces was collected and weighed per stool. Sample pre-treatment involved diluting and homogenizing the feces by adding water (1:3 *w*/*v*; B. Braun). A T25 basic Ultra Turrax (IKA Works, Staufen, Germany) was used to homogenize the samples before aliquoting. Urine and feces sample collection continued until at least 90% of the administered radioactivity was excreted in urine plus feces, or < 1% of excretion in urine plus feces per 24 h interval on at least 2 consecutive days was detected.

#### Intracellular pharmacokinetics and bioactivation

Peripheral blood mononuclear cells (PBMCs) were collected 24 h after day 5 dosing of ^14^C-guadecitabine for the exploratory analysis of the intracellular pharmacokinetics of ß-decitabine. For each patient, a single sample was collected according to the previously reported procedures. [11] In short, PBMCs were isolated from 20 mL of THU-stabilized whole blood using Ficoll density gradient. After isolation and washing of the PBMCs. a cell count was performed and PBMCs were then lysed using methanol. In the obtained lysate, intracellular decitabine nucleotides were quantified as the amount of decitabine-triphosphate (DEC-TP), as well as the total amount of decitabine related material in the cell (DEC-XP), using two previously developed and validated LC-MS/MS assays. [11, 12]

In addition, the amount of genomic DNA incorporated ß-decitabine was analyzed in selected whole blood samples. To achieve this, genomic DNA was extracted from 400 μL of THU-stabilized whole blood. DNA was then enzymatically digested into single nucleosides and analyzed for ß-decitabine content using a previously validated LC-MS/MS assay. [11]

### Total radioactivity analysis

Total radioactivity analysis was performed on a Liquid Scintillation Counter Tri-Carb 2910 (PerkinElmer, Groningen, The Netherlands). Aliquots of 200 μL of whole blood, plasma, plasma-ultrafiltrate and feces homogenate (all in duplicate), and 1 mL of urine (in singular) were transferred to scintillation vials. Isopropanol (1 mL) was added to fecal samples to dissolve fibers, 0.5 M potassium ethylenediaminetetra-acetic acid (NaEDTA) pH 8.0 (0.1 mL) was added to whole blood to reduce foaming, and Solvable™ (1 mL) was added to both matrices to solubilize tissue. Hydrogen peroxide 30% (*w*/w) (0.4 mL to feces and 0.5 mL to whole blood) was added to reduce color intensity. These samples were placed in a water bath (40 °C, GF1086, Salmen Kipp, Breukelen, The Netherlands) to stimulate the chemical processes. Finally, 10 mL of Ultima Gold™ Cocktail (PerkinElmer) was added and the samples were analyzed, either for 60 min or until the 2-sigma error was less than or equal to 5%, whichever came first. A scintillate blank was used to provide as a background sample. Results were expressed as a percentage of the administered radioactive dose for urine and feces and in guadecitabine equivalents for whole blood, plasma, and plasma ultrafiltrate.

### Quantification of guadecitabine and ß-decitabine in plasma, whole blood and urine

Plasma, whole blood and urine samples collected up to 144 h after administration were analyzed for guadecitabine and ß-decitabine concentrations using validated LC-MS/MS assays. [13] Routine sample analysis acceptance criteria for bioanalytical data according to FDA and EMA guidelines [14, 15] were applied and results were reported using Analyst 1.6.2. software (Sciex).

### Pharmacokinetic analysis

Guadecitabine and ß-decitabine plasma concentrations were used to determine the maximum observed plasma concentration (C_max_), time to reach maximum plasma concentration (T_max_), area under the plasma concentration-time curve from time zero to time of the last measurable concentration (AUC_0-last_) and to infinity (AUC_0-inf_), the terminal phase half-life (t½) and the elimination rate constant from the central compartment (k_e_). The apparent volume of distribution during the terminal phase (V_z_/F) and the apparent total clearance of the drug from plasma (CL/F) were calculated for guadecitabine only. Parameters were calculated using pharmacokinetic curves obtained on Day 1 and Day 5 of cycle 1. Non-compartmental analysis was performed using R version 3.0.1. [16]

### Safety assessment

The safety of guadecitabine was evaluated during the complete study by monitoring adverse events (AEs), clinical laboratory, vital signs, 12-lead electrocardiogram (ECG) and physical examination. The National Cancer Institute Common Terminology Criteria for Adverse Events (NCI CTCAE) grading system was used to describe the severity of the AEs that occurred.

### Metabolite profiling

#### Sample selection

Urine samples up to 48 h were used for metabolite profiling based on the radioactivity excretion profile observed. Intrapatient pooling was performed based on the weight of the individual samples. The samples within 0–24 h and 24–48 h time period were pooled for each patient. Based on the observed radioactivity in the plasma samples, only samples around the C_max_ of radioactivity analysis were used for metabolite profiling. For each patient, a plasma sample at 2.5 h post-dosing was used for metabolite profiling.

#### Sample preparation

For both plasma and urine, 300 μL aliquots were mixed with 900 μL methanol. 900 μL of the obtained supernatant was evaporated to dryness under a gentle stream of nitrogen at 40 °C. The dried supernatant was reconstituted in methanol/water (80:20, *v*/v %). An aliquot of the clear supernatant was analyzed for radioactivity to calculate the pre-treatment recovery. The remaining supernatant was used for metabolite identification and quantification.

#### LC-MS-LSC systems

Selected plasma and pooled urine samples were analyzed by liquid chromatography coupled to a mass spectrometer in combination with off-line liquid scintillation counting (LC-MS-LSC). Plasma radioactivity concentrations were too low to obtain accurate mass spectra with an Orbitrap high-resolution mass spectrometer. For this reason, a more sensitive triple quadrupole mass spectrometer was selected as a detector and mass transitions that were identified during the urine metabolite profiling were monitored. Separate methods were used for urine and plasma analysis (Orbitrap high-resolution mass spectrometry for urine and QTRAP5500 triple quadrupole mass spectrometer for plasma). Details on both methods can be found in Supplementary Table 1.

#### Metabolite identification and quantification

To separate metabolites bearing the ^14^C-label, a 60-min liquid chromatography gradient was applied. Using a post-column flow splitter, the eluate was directed to a fraction collector as well as to the mass spectrometer (3:1, *v*/v ratio). Fractions of 1 min. Were collected with the fraction collector and the radioactivity in each fraction was measured using liquid scintillation counting. Semi-quantitative determinations of guadecitabine metabolites in urine were performed based on the obtained total radioactivity counts from each fraction, after correction for injection volume, split ratio, sample pretreatment, and processed sample volume. Identification of the metabolites was based on the accurate Orbitrap MS, MS^2^ and MS^3^ data for urine analysis, and multiple reaction monitoring for plasma analysis, in the fractions where radioactivity was observed.

## Results

### Patient demographics and safety

In total, five patients with solid tumors were enrolled in the mass balance trial. An overview of patient baseline characteristics can be found in Table 1. The patients who participated in this study experienced a total of 63 adverse events, of which 32 events were judged to be related to guadecitabine. Adverse events with the highest incidence were neutropenia (9 events) and fatigue (6 events), in four patients each. Two adverse events of neutropenia and one adverse event of increased gamma-glutamyltransferase were of CTCAE grade 4. One serious adverse event occurred (vomiting), which was not judged to be related to guadecitabine. The overall safety and tolerability profile of guadecitabine was considered acceptable given the clinical background of the study population.

**Table 1:** Patient baseline characteristics

Characteristic	Number of patients
Total number of patients (%)	5 (100)
Sex	
Male (%)	3 (60)
Female (%)	2 (40)
Ethnic origin	
Caucasian (%)	5 (100)
Age	
Median (range), years	59 (54–73)
Weight	
Median (range), kg	63.3 (58.0–93.7)
Height	
Median (range), cm	171 (166–187)
WHO performance status	
0 (%)	2 (40)
1 (%)	3 (60)
Primary tumor type (%)	
Colorectal (%)	3 (60)
Sarcoma (%)	1 (20)
Melanoma (%)	1 (20)
Administered dose	
Median (range), mg	75.9 (71.4–93.4)
Median (range), μCi	71.1 (66.0–87.8)

*WHO* World Health Organization

### Mass balance of ^14^C-guadecitabine

**Fig. 2:**
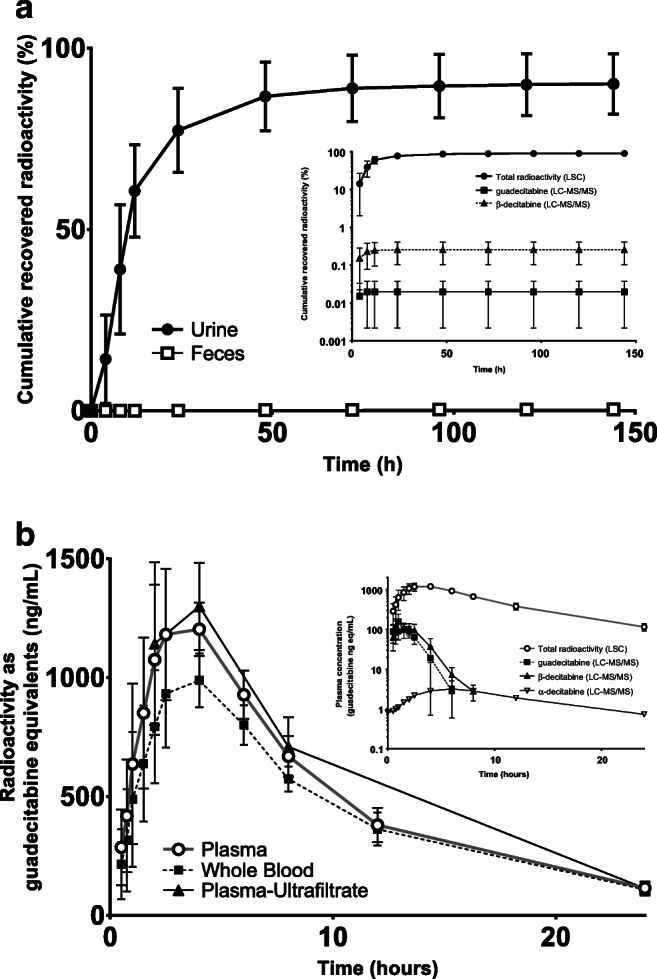
**a** Cumulative recovered radioactivity in excreta (mean ± SD) after a single dose of ^14^C-guadecitabine in patients with advanced cancer (*n* = 5) and **(a, insert)** the log-linear cumulative recovery of total radioactivity (as analyzed by liquid scintillation counting (LSC)), guadecitabine and ß-decitabine (as analyzed by LC-MS/MS) in urine (mean ± SD) in the same five patients. **b** Concentration-time profile (mean ± SD) of total radioactivity in whole blood, plasma, and plasma ultrafiltrate and **(b, insert)** the log-linear concentration-time profile of total radioactivity (as analyzed by LSC), guadecitabine, ß-decitabine, and α-decitabine (as analyzed by LC-MS/MS) in plasma (mean ± SD) after a single dose of ^14^C-guadecitabine to patients with advanced cancer (*n* = 5)

The cumulative recovered radioactivity in urine and feces is visualized in Fig. 2a (individual patient data displayed in Supplementary Table 2). The total excretion of radioactivity up to the last point of collection ranged from 77.6% to 98.4%, with a mean of 90.5%. Radioactivity was almost exclusively excreted in urine, with virtually no excretion in feces. After administration of ^14^C-guadecitabine, the excretion of radioactivity was rapid, with a mean recovery of 77.3% within 24 h and 86.8% within 48 h after administration.

Both guadecitabine and ß-decitabine are almost completely metabolized prior to excretion, with negligible amounts of unchanged drug in urine for both compounds (Fig. 2a, insert). Guadecitabine and ß-decitabine excretion in urine only accounted for 0.02% and 0.3% of the mean administered dose, respectively, as quantified by LC-MS/MS. The radioactivity in urine is therefore almost completely explained for by metabolites bearing the radioactive ^14^C-label.

### ^14^C-Guadecitabine in the systemic circulation

Figure 2b demonstrates the concentration-time profile of radioactivity in the systemic circulation after the subcutaneous administration of ^14^C-guadecitabine. After administration, radioactivity is absorbed into the systemic circulation, with a maximum radioactivity concentration 4 h after administration. Radioactivity is rapidly eliminated from the systemic circulation in a biphasic manner, with no detectable concentrations later than 24 h after drug administration. The radioactive curves for plasma, whole blood and plasma ultrafiltrate indicate that there was almost no protein or erythrocyte binding or distribution into red blood cells from the circulating radioactive material, which was also seen for guadecitabine and ß-decitabine. [13] By converting the total radioactivity in plasma to guadecitabine equivalents, the contribution of guadecitabine and ß-decitabine (as quantified using LC-MS/MS) to the total radioactivity observed in plasma was calculated. As visualized in Fig. 2b (insert), ß-decitabine and guadecitabine only accounted for 8.1% and 5.4% of total radioactivity present in plasma collected 2.5 h after ^14^C-guadecitabine administration, respectively. There was a large and increasing gap between the total radioactivity present in plasma and the guadecitabine and ß-decitabine curves, which is explained by the formation of metabolites bearing the ^14^C label. The elimination half-life for the radioactivity curve is flattened as compared to the guadecitabine and ß-decitabine curves, indicating that there was longer retention of radioactive metabolites compared to guadecitabine or primary metabolite ß-decitabine.

### Pharmacokinetics of guadecitabine and ß-decitabine in plasma

A summary of pharmacokinetic parameters of guadecitabine and ß-decitabine in plasma on Day 1 and Day 5 can be found in Supplementary Table 3. Guadecitabine reached a mean C_max_ approximately 2 h after dosing on Day 1 and approximately 1 h after dosing on Day 5. Plasma drug exposures on Day 1 and Day 5 of therapy were similar, with a mean AUC_0-inf_ of 250 ng·h/mL on Day 1 and a mean AUC_0-inf_ of 277 ng·h/mL on Day 5, indicating that there is no drug accumulation of guadecitabine in the circulation during a treatment cycle.

Guadecitabine displayed a terminal elimination half-life of less than 1 h, indicating rapid conversion to the active metabolite ß-decitabine and other metabolites. ß-Decitabine reached mean C_max_ levels approximately 2 h after administration of guadecitabine on Day 1 and Day 5. The mean C_max_ concentrations were 42.9 ng/mL and 51.4 ng/mL on Day 1 and Day 5, respectively. ß-Decitabine was rapidly metabolized and eliminated from the systemic circulation, with a terminal elimination half-life of around 1 h. Guadecitabine and ß-decitabine plasma concentrations were quantifiable up to 8 h after administration. The AUC_0-tlast_ covers more than 95% of the AUC_0-inf_ indicating that sufficient samples were collected to extrapolate the total exposure for both guadecitabine and ß-decitabine.

### Metabolite profiling

Collected urine samples demonstrated much higher radioactivity than plasma samples, as can be explained by the rapid elimination from the systemic circulation and subsequent excretion in urine. Based on the very low recoveries of both guadecitabine and ß-decitabine in this matrix, it was concluded that the radioactivity found in urine was almost exclusively accounted for by metabolites. For these reasons, metabolite profiling in urine was performed prior to the metabolite profiling in plasma.

As the mean radioactivity excreted in feces up to the last point of collection was trivial at only 0.4% (Fig. 2a), metabolite profiling was not conducted for feces.

#### Metabolites in urine

A mean of 86.7% of the administered radioactivity was recovered in urine in the first 48 h after the administration of ^14^C-guadecitabine (Supplementary Table 2). Based on this rapid urinary excretion, only the samples collected up to this time point were analyzed and screened for the presence of metabolites. Samples collected within the first 24 h after administration of ^14^C-guadecitabine were pooled (intrapatient) based on the weight of excreted urine per time point. The pooling accuracy of urine samples was between 98% and 112% for all patients based on radioactivity counts. The radioactive recovery after sample-pretreatment was between 79% and 104%.

**Fig. 3:**
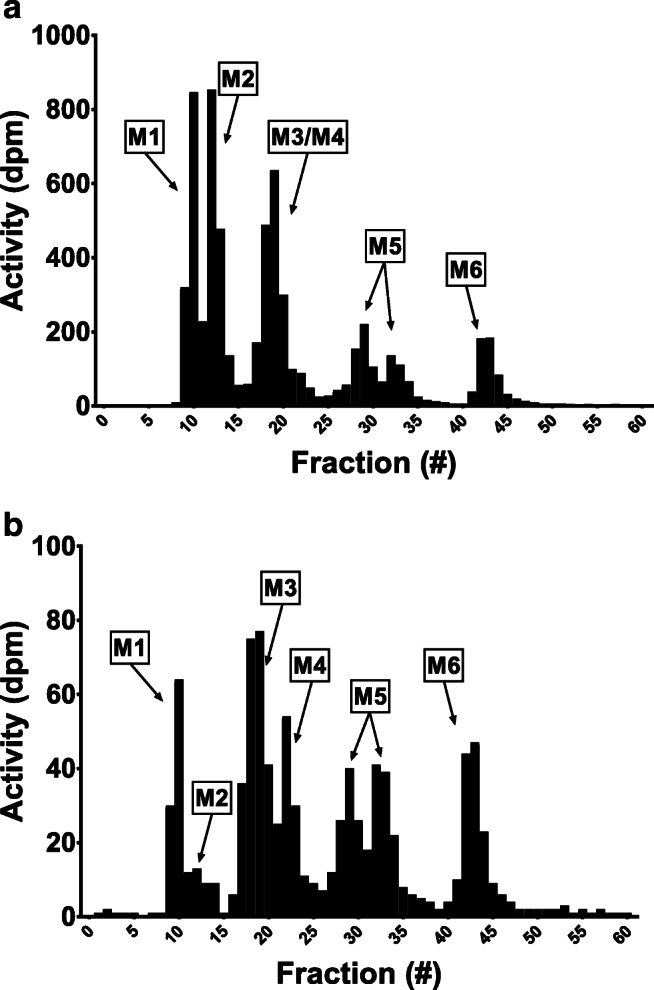
Representative radiochromatograms of a patient urine sample collected from (**a**) 0-24 h and (**b**) 24-48 h after administration of ^14^C-guadecitabine to patients with advanced cancer (n = 5). Fractions related to metabolites 1–5 (M1-M5) are indicated. Note that the scale of the y-axis is different in each radiochromatogram

Representative radiochromatograms from pooled urine samples collected from a single patient from 0 to 24 h and 24–48 h after subcutaneous ^14^C-guadecitabine administration are shown in Fig. 3. Different fractions display radioactivity, which suggests the presence of different metabolites bearing the radioactive label(s). In total, six different peaks were identified and coded M1 to M6 based on their elution time. All identified peaks were present both in the 0–24 h and 24–48 h samples, and radiochromatograms between individual subjects demonstrated a low variability, indicating similar metabolite profiles for each patient. The combined sum of radioactivity in the fractions almost equaled total radioactivity present in the sample, showing that all radioactivity was accounted for in the radiochromatogram. Guadecitabine and ß-decitabine levels were below the limit of detection for the radioactivity analysis and were therefore not present in the radiochromatograms.

#### Metabolites in plasma

For each patient, a single time point of 2.5 h was used to identify radioactive metabolites present in the systemic circulation. The sample-pretreatment recovery of total radioactivity in the processed plasma samples was between 74% and 87%. Plasma radiochromatograms for each patient demonstrated that at 2.5 h post-dose, metabolite concentrations were only just above the limit of detection for radioactivity analysis. The radioactive peaks in the plasma radiochromatograms were observed at the same retention times as for urine, indicating that the same metabolites might be present in the systemic circulation.

### Metabolite identification in urine

For M1 to M5, masses were observed that could be potentially linked to guadecitabine metabolites. These masses were used to identify and confirm metabolite structures using high-resolution mass spectrometry data of the mass fragmentation patterns. High-resolution mass spectrometry data were obtained using both the positive ionization and the negative ionization mode. The structural characterization of the observed metabolites is described below. An overview of metabolite characteristics, including fragmentation patterns and accurate mass confirmations, can be found in Supplementary Table 4. The structure of metabolite 6 (M6) was not elucidated.

#### M1

Metabolite 1 (M1) has a deprotonated parent mass of 129 Da and is proposed to be formed out of M2 after removal of the ribose group of the ß-decitabine structure (nucleoside to nucleobase conversion).

#### M2

Metabolite 2 (M2) has a deprotonated parent mass of 245 Da and is proposed to be formed out of ß-decitabine after oxidative deamination and additional carbon oxidation, both in the nucleobase part of the structure.

#### M3

Metabolite 3 (M3) has a deprotonated parent mass of 247 Da and is proposed to be formed after oxidative deamination and hydrolysis of the nucleobase part of the ß-decitabine molecule.

#### M4

Metabolite 4 (M4) has a deprotonated parent mass of 219 Da and is proposed to be formed after deformylation of M3, where the formyl group is cleaved off after hydrolytic ring opening.

#### M5

It is proposed that metabolite 5 (M5) has a protonated parent mass of 218 Da and is formed after hydrolysis and deformylation of ß-decitabine. There were 2 peaks visible for M5 in both the radiochromatogram and high-resolution mass spectrometry chromatograms. These peaks could be explained for by an α and ß form of M5, formed by anomerization of the carbon-nucleobase bond in the structure.

#### ß-Decitabine isomers

**Fig. 4:**
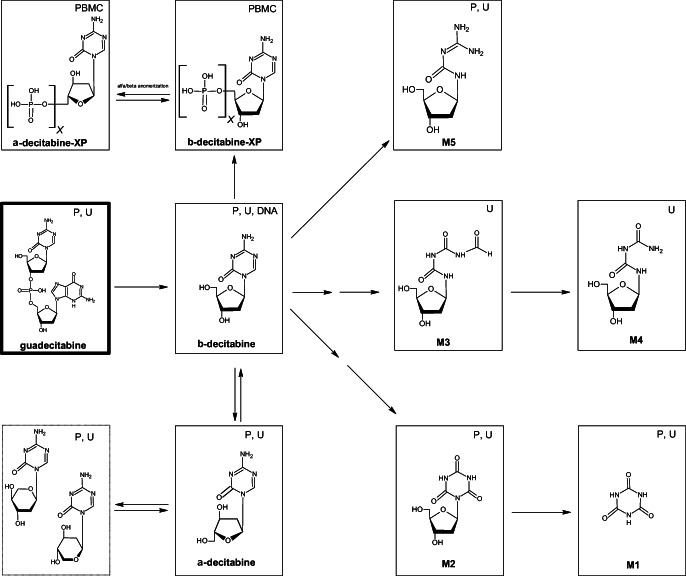
Proposed metabolic pathway of ^14^C-guadecitabine (detected in: P = Plasma, U = Urine, PBMC = peripheral blood mononuclear cell, DNA = deoxyribonucleic acid)

During the quantification of ß-decitabine in urine using LC-MS/MS, additional peaks were observed with the same parent and fragment mass as for ß-decitabine (Supplementary Fig. 1). [13] These peaks are thought to be formed by ß- to α-decitabine anomerization of the carbon-nucleoside bond and by furanoside to pyranoside conversion of the sugar ring, as previously described for similar nucleoside structures (Fig. 4, dashed lines). [13, 17, 18] The presence of α-decitabine in urine could be confirmed using a stable isotope-labeled internal standard of decitabine, containing a mixture of α- and ß-decitabine (Supplementary Fig. 1).

### Metabolite identification in plasma

Due to the low metabolite concentrations present in plasma, radioactive peaks that were visible in the radiochromatograms could not be confirmed using high-resolution mass spectrometry analysis. Based on the identified metabolites in urine, the fragmentation patterns of M1-M5 were used to quantify these metabolites in plasma using a more sensitive LC-triple quadrupole MS/MS approach. M1, M2 and M5 responses (based on *m/z* transitions) could be measured and identification of these metabolites was performed based on their retention times (comparison with the retention times of the metabolites identified in the urine samples) and multiple reaction monitoring (MRM) transitions. Confirmation of M3 and M4 in plasma was not possible due to concentrations being too low for detection and there was insufficient selectivity to distinguish the metabolites from other (endogenous) molecules with the same mass transitions.

### Metabolite quantification

**Fig. 5:**
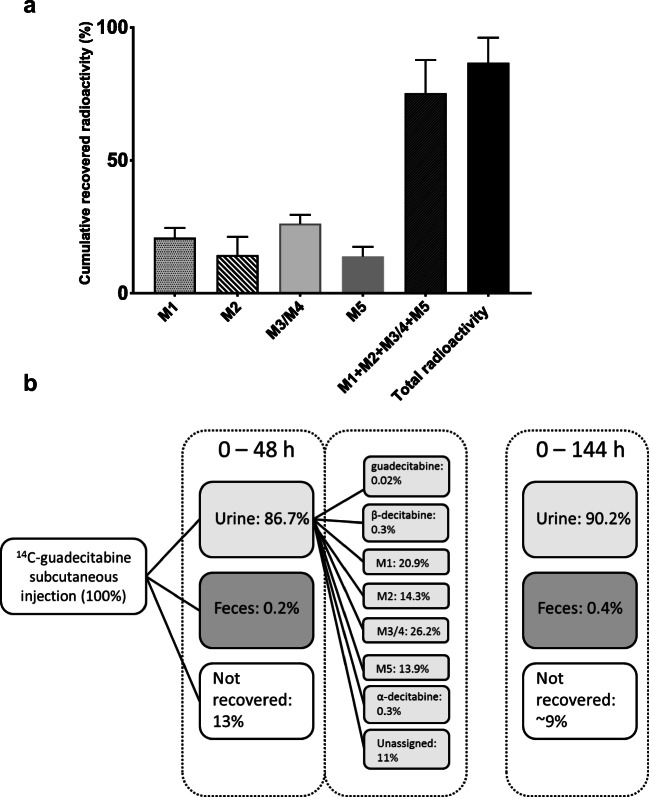
(**a**) Cumulative recovered radioactivity of metabolites found in urine (mean ± SD) between 0 and 48 h after a single dose of ^14^C-guadecitabine to patients with advanced cancer (n = 5) and (**b**) Summary of guadecitabine metabolite excretion after a single subcutaneous administration of ^14^C-guadecitabine. The figure shows the recovery of radioactivity in feces and urine (up to 48 h and 144 h) and the excretion of metabolites up to 48 h after a single subcutaneous injection of ^14^C-guadecitabine

For the five identified metabolites in urine, concentrations of each metabolite were calculated based on the radioactivity counts as observed in the radiochromatograms and were expressed as a percentage of the administered radioactive dose. A summary of the quantification of the urine metabolites found in the radiochromatograms is visualized in Fig. 5a (individual patient data shown in Supplementary Table 5). Within 48 h after subcutaneous administration of ^14^C-guadecitabine, 86.7% of the radioactivity is recovered in urine. Over this time course, the identified metabolites were excreted in similar amounts, with a combined total that approximated the total amount of radioactivity excreted. M1-M5 therefore almost completely explain the radioactivity that was excreted in urine. Adding M6, the total radioactivity was completely accounted for. M3 and M4 were not baseline-separated in the chromatograms and therefore their combined contribution to the mean administered dose after 48 h is reported. α-Decitabine excretion in urine was quantified using LC-MS/MS and accounted for 0.3% of the total administered dose. Other ß-decitabine isomers were even less abundant and therefore considered insignificant.

Concentrations of the identified metabolites in plasma (M1, M2, and M5) were too low to quantify based on the radioactivity that was found in the collected fractions. It was possible to detect and perform a semi-quantitation of α-decitabine by reprocessing results obtained for ß-decitabine analysis by LC-triple quadrupole MS/MS (Fig. 2b, insert). α-Decitabine displays a prolonged terminal elimination half-life as compared to ß-decitabine that is in line with the observed terminal elimination half-life for the total radioactivity. However, concentrations and total exposure were significantly lower than for ß-decitabine.

### Intracellular pharmacokinetics and bioactivation

Intracellular decitabine triphosphate concentrations were quantified in five treated patients, 24 h after ^14^C-guadecitabine administration. At this point, both guadecitabine and ß-decitabine were eliminated from the systemic circulation (Fig. 2b, insert). Decitabine triphosphate levels were detectable in all five patients at corrected concentrations ranging from 32.7–158 fmol / million PBMCs using the assay by Jansen et al. [12] These results are in line with previously reported results after ß-decitabine intravenous infusion, where a prolonged intracellular effect was observed as compared to the systemic exposure to ß-decitabine. [12, 19] Using an anomer-selective LC-MS/MS assay, α-decitabine nucleotides were also observed in patient samples, indicating that both anomers are present inside the PBMCs (Fig. 4). [11]

In the first four included patients, the degree of ß-decitabine incorporation in genomic DNA isolated from whole blood was investigated. Whole blood samples from predose, Day 1 (2 h post-injection), Day 5 (2 h post-injection), and Day 6 (24 h post Day 5 injection) of cycle 1 were analyzed for ß-decitabine DNA incorporation. ß-decitabine incorporation levels were detectable in all four patients 24 h post the last dose, indicating a prolonged intracellular effect that is in line with the observed ß-decitabine triphosphate concentrations at this time point. [11] α-Decitabine DNA incorporation was not detected in any of the patient samples, providing confirmation that only ß-decitabine is incorporated into the DNA. [11]

## Discussion

A summary of ^14^C-guadecitabine metabolite excretion after a single subcutaneous injection of ^14^C-guadecitabine can be found in Fig. 5b. Guadecitabine was rapidly metabolized and excreted in urine, with a trivial amount of excretion of radioactivity in feces. Both guadecitabine and ß-decitabine demonstrated very low excretion as unchanged drug in urine (0.02% for guadecitabine and 0.3% for ß-decitabine). This is in line with data on the excretion of ß-decitabine after intravenous administration, where <1% of decitabine was excreted in urine as unchanged drug. [20]

The proposed metabolic pathway of ^14^C-guadecitabine can be found in Fig. 4. The identified metabolites accounted for 86.9% of the recovered radioactivity from 0 to 48 h after drug administration. All identified metabolites were ß-decitabine related products, suggesting efficient conversion via almost complete cleavage of the phosphodiester bond between ß-decitabine and deoxyguanosine prior to elimination. The identified metabolites were formed by processes of oxidation, oxidative deamination, hydrolysis and combinations thereof in the ß-decitabine part of the structure. As the binding of ß-decitabine to deoxyguanosine results in the inability of cytidine deaminase to oxidize the amino group in the ß-decitabine structure [2], metabolites that have undergone oxidative deamination were likely formed after the release of ß-decitabine from the guadecitabine structure (M1-M4). Metabolites formed by ring-opening of the pyrimidine ring and conversion of the sugar ring (isomerization) are both a result of hydrolysis. Metabolite M5 was previously confirmed as a ß-decitabine decomposition product [21, 22] but was not yet confirmed as an in vivo formed metabolite. Metabolites M3 and M4 follow these same processes, but only after prior oxidative deamination of the pyrimidine ring. M1-M5 are all thought to be inactive because of structural alterations in the reactive nitrogen side in the pyrimidine ring of the ß-decitabine structure. Based on these results, additional research in patients with renal and/or hepatic impairment is considered less relevant.

ß-Decitabine undergoes beta to alpha anomerization in the systemic circulation. In addition to α- and ß-decitabine, additional peaks in plasma and urine chromatograms that were not explained for by α- and ß-decitabine were found. [13] These peaks are hypothesized to be caused by furanoside to pyranoside conversion of the sugar ring, resulting in additional isomers with the same *m/z* ratio as α- and ß-decitabine. It is known that deoxyribose can be interconverted to multiple isoforms at physiological conditions, and it is therefore hypothesized that α- and ß-decitabine are converted to these isoforms in-vivo as well. [17, 22, 23] Using radioactivity analysis, these structures could not be identified, as the proportional presence of ß-decitabine itself and its isomers were a negligible part of the excreted metabolites in urine and was below the limit of quantification. However, the identification of these structures using a more sensitive LC-MS/MS approach gave additional insight into the metabolic pathway of guadecitabine and ß-decitabine.

ß-Decitabine is phosphorylated intracellularly to its ultimate active metabolite ß-decitabine-triphosphate. It was demonstrated that after guadecitabine administration, this ultimate active metabolite is indeed found inside PBMCs. [11] It was also demonstrated that ß-decitabine, and not α-decitabine, was incorporated into DNA, despite the intracellular presence of α-decitabine nucleotides. ß-decitabine DNA incorporation accumulates during a guadecitabine treatment cycle, with detectable DNA incorporation 24 h post-dosing. Intracellular levels of ß-decitabine triphosphate and DNA incorporated ß-decitabine may serve as a potential new marker to monitor treatment efficacy, by enabling drug quantification at the DNA target level.

In conclusion, the mass balance and metabolite profiling study of ^14^C-guadecitabine accomplished all its objectives, and characterized the pathway of guadecitabine and its metabolites throughout the body after subcutaneous guadecitabine administration in cancer patients, with consistent results seen in samples analyzed from all 5 subjects from this trial.

## Electronic supplementary material

ESM 1 (PDF 539 kb)
