# Supplementary material for: Mass balance and metabolite profiling of 14C-guadecitabine in patients with advanced cancer
Source: Invest New Drugs. 2019 Oct 11;38(4):1085–95. doi: 10.1007/s10637-019-00854-9 (PMC7340650; doi:10.1007/s10637-019-00854-9)

Supplementary Tables and Figures

Supplementary Table 1. HPLC and mass spectrometry settings of the Orbitrap high resolution mass spectrometry system and the QTRAP 5500 LC-MS/MS system

Supplementary Table 2. Radioactive recovery in urine and feces after subcutaneous administration of ^{14}C-guadecitabine from 0-48h and up to the last point of collection

Supplementary Table 3. Summary of pharmacokinetic parameters of guadecitabine and ß-decitabine in plasma on Day 1 and Day 5 after subcutaneous administration of guadecitabine at a dose of 45 mg/m^2

Supplementary Table 4. Proposed metabolite structures and fragmentation patterns of identified metabolites in urine using Orbitrap accurate mass analysis

Supplementary Table 5. Cumulative recovery of guadecitabine metabolites in urine (mean ± SD) from 0-24h and 0-48h, based on total radioactivity measurements following administration of subcutaneous ^{14}C-guadecitabine at a dose of 45 mg/m^2 (n=5)

Supplementary Figure 1. Multiple reaction monitoring chromatograms of a patient urine sample collected approximately **(A)** 4 h, and **(B)** 48 h after administration of subcutaneous guadecitabine 45 mg/m^2 monitoring **(A, B)** decitabine (——— *m/z* 229.1 → 113.1) and **(C, D)** decitabine-IS (------ *m/z* 235.1 → 119.1) mass transitions.

Supplementary Table 1.

**Pump**	**LC-10AD (Shimadzu)**		**Acquity I Class (Waters)**		
**Flow rate**	1.0 mL/min		1.0 mL/min		
**Analytical column**	Luna NH2 (250 x 4.6 mm, 5 µm)		Luna NH2 (250 x 4.6 mm, 5 µm)		
**Column oven**	35 °C		Acquity I Class (Waters)		
**Run time**	60 min		60 min		
**Mobile phase A**	IPA: 1M ammonium formate, pH 3.0: ACN (12:0.24:88, v/v/v)		IPA: 1M ammonium formate, pH 3.0: ACN (12:0.24:88, v/v/v)		
**Mobile phase B**	IPA: 1M ammonium formate, pH 3.0: ACN (49:1:50, v/v/v)		IPA: 1M ammonium formate, pH 3.0: ACN (49:1:50, v/v/v)		
**Gradient composition**	Mobile phase B: 20% (0-3 min), 20% → 100% (3-28 min), 100% (28-55 min), 100% →20% (55-55.1 min), 20% (55.1-60 min)		Mobile phase B: 20% (0-3 min), 20% → 100% (3-28 min), 100% (28-55 min), 100% →20% (55-55.1 min), 20% (55.1-60 min)		
**Autosampler**	**SIL HTc (Shimadzu)**		**Acquity I Class (Waters)**		
**Tray temperature**	15 °C		5 ± 3 °C		
**Injection volume**	50 µL		10 µL		
**Splitter**	**Accurate (LC packings)**		**Accurate (LC packings)**		
**Split ratio (collector:MS)**	3:1 (v/v)		3:1 (v/v)		
**Mass spectrometer**	**LTQ Orbitrap (Thermo)**		**QTRAP 5500 (AB Sciex)**		
**Ionization/interface**	ESI, positive ionization mode	ESI, negative ionization mode	**Ionization/interface**	ESI, positive ionization mode	ESI, negative ionization mode
**Scan range (m/z)**	50-1000	50-1000	**Monitored transitions (m/z)**	558.1 → 446.0 (SGI-110), 229.1 → 113.0 (DEC), 219 → 103.0 (M5)	128.0 → 85.0 (M1), 244.1 → 85.0 (M2), 246.1 → 129.0 (M3), 218.0 → 102.0 (M4)
**Monitored transitions (m/z)**	NA	NA	**Ionspray voltage (V)**	5500	-4500
**Sheath gas flow (au)**	25	25	**Ion source gas 1 (au)**	50	50
**Aux gas flow (au)**	5	5	**Ion source gas 2 (au)**	50	50
**Sweep gas flow (au)**	2	2	**Curtain gas (au)**	30	30
**Spray voltage (kV)**	3.5	5	**Collision gas (au)**	8	8
**Normalized collision energy (V)**	35	40	**Temperature (°C)**	400	400
**Capillary temperature (°C)**	300	350	**Dwell time (ms)**	30	30
**Capillary voltage (V)**	35	-22	**Collision energy (V)**	20	-20
**Tube lens (V)**	52	-43	**Collision exit potential (V)**	10	-10
			**Declustering potential (V)**	125	-125

Supplementary Table 2.

	0-48 h			Up to the last point of collection			
**Patient (#)**	**Urine (%)**	**Feces (%)**	**Total (%)**	**Urine (%)**	**Feces (%)**	**Total (%)**	**Collection period (h)**
**1**	72.3	0.0	72.3	77.4	0.3	77.6	144
**2**	91.3	0.0	91.3	95.5	0.4	95.8	144
**3**	82.1	0.2	82.4	86.6	0.5	87.1	144
**4**	95.6	0.2	95.7	98.2	0.2	98.4	72
**5**	92.2	0.2	92.4	93.2	0.4	93.6	72
**Mean**	86.7	0.1	86.8	90.2	0.4	90.5	NA
**SD**	8.5	0.1	8.5	7.5	0.1	7.4	NA

Supplementary Table 3.

Parameter		Guadecitabine Day 1 (n=5)	Guadecitabine Day 5 (n=5)	ß-decitabine Day 1 (n=5)	ß-decitabine Day 5 (n=5)
**C_{max} (ng/mL)**	Mean	108	165	42.9	51.4
	CV (%)	43.6	46.1	35.1	28.4
**T_{max} (h)**	Mean	1.85	0.90	2.05	1.80
	CV (%)	75.0	24.8	61.2	31.7
**$AUC_{0\text{-}last}$ (ng·h/mL)**	Mean	245	269	130	134
	CV (%)	22.0	28.7	31.7	22.0
**$AUC_{0\text{-}inf}$ (ng·h/mL)**	Mean	249	275	135	136
	CV (%)	22.0	26.4	33.0	21.9
**t½ (h)**	Mean	0.78	0.67	1.12	0.95
	CV (%)	37.8	36.8	30.4	7.39
**k_e (h^{-1})**	Mean	1.04	1.14	0.68	0.73
	CV (%)	48.3	35.7	34.2	7.91
**V_z/F (L/m^2)**	Mean	204	172	NA	NA
	CV (%)	31.8	49.7	NA	NA
**CL/F (L/h/m^2)**	Mean	189	172	NA	NA
	CV (%)	26.6	23.8	NA	NA

NA Not applicable
$AUC_{0\text{-}inf}$ Area under the plasma concentration-time curve from time 0 to infinity, *$AUC_{0\text{-}last}$* Area under the plasma concentration-time curve from time 0 to the last quantifiable concentration, *CL/F* Apparent oral clearance, *C_{max}* maximum observed plasma concentration, *CV* Coefficient of variation, *T_{max}* time to reach maximum observed plasma concentration, *t½* terminal half-life, *V_z/F* Apparent volume of distribution during terminal phase

Supplementary Table 4.

Metabolite	Proposed structure and fragmentation	Retention time (min)	MS	Theoretical monoisotopic ion mass (Da)	Measured monoisotopic ion mass (Da)	Δppm
**M1**	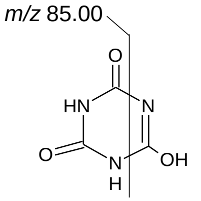	14-16	MS	128.01016	128.01019	0.234356
			MS^2	85.00435	85.00456	2.470462
**M2**	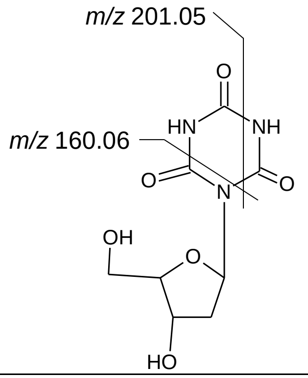	10.5-12.5	MS	244.05751	244.05733	-0.737531
			MS^2	201.05169	201.05139	-1.492154
			MS^2	160.06153	160.06157	0.249904
**M3**	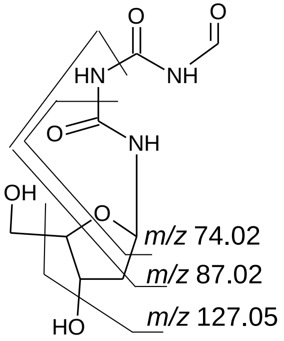	14-16	MS	246.07316	246.07290	-1.056596
			MS^2	127.05130	127.05122	-0.629667
			MS^2	87.02000	87.02008	0.919329
			MS^3	74.02475	74.02493	2.431619

**M4**	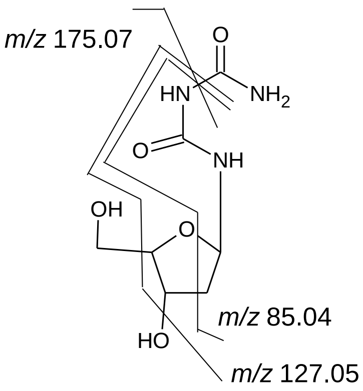	16-20	MS	218.07824	218.07828	0.183420
			MS^2	175.07243	175.07228	-0.856788
			MS^3	85.04074	85.04095	2.469405
			MS^3	127.05130	127.05129	-0.078708
**M5**	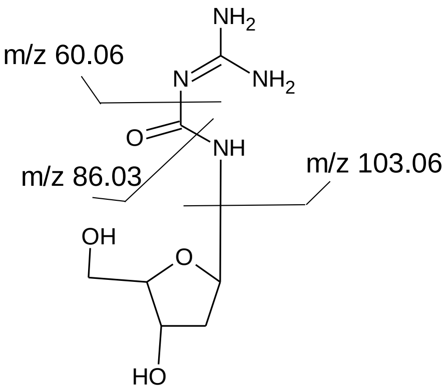	25-35	MS	219.10878	219.10840	-1.734298
			MS^2	175.07243	175.07228	-0.856788
			MS^3	85.04074	85.04095	2.469405
			MS^3	127.05130	127.05129	-0.078708

Supplementary Table 5.

	% of administered dose	
**Metabolite**	*0-24h*	*0-48h*
**M1**	19.6 (3.8)	20.9 (3.7)
**M2**	14.0 (6.8)	14.3 (6.8)
**M3/4**	23.0 (4.0)	26.2 (3.3)
**M5**	11.8 (3.4)	13.9 (3.6)
**Total of M1-M5**	**68.3 (14.1)**	**75.3 (12.5)**
**Total radioactivity**	**77.3 (11.6)**	**86.7 (9.5)**

Supplementary Figure 1.

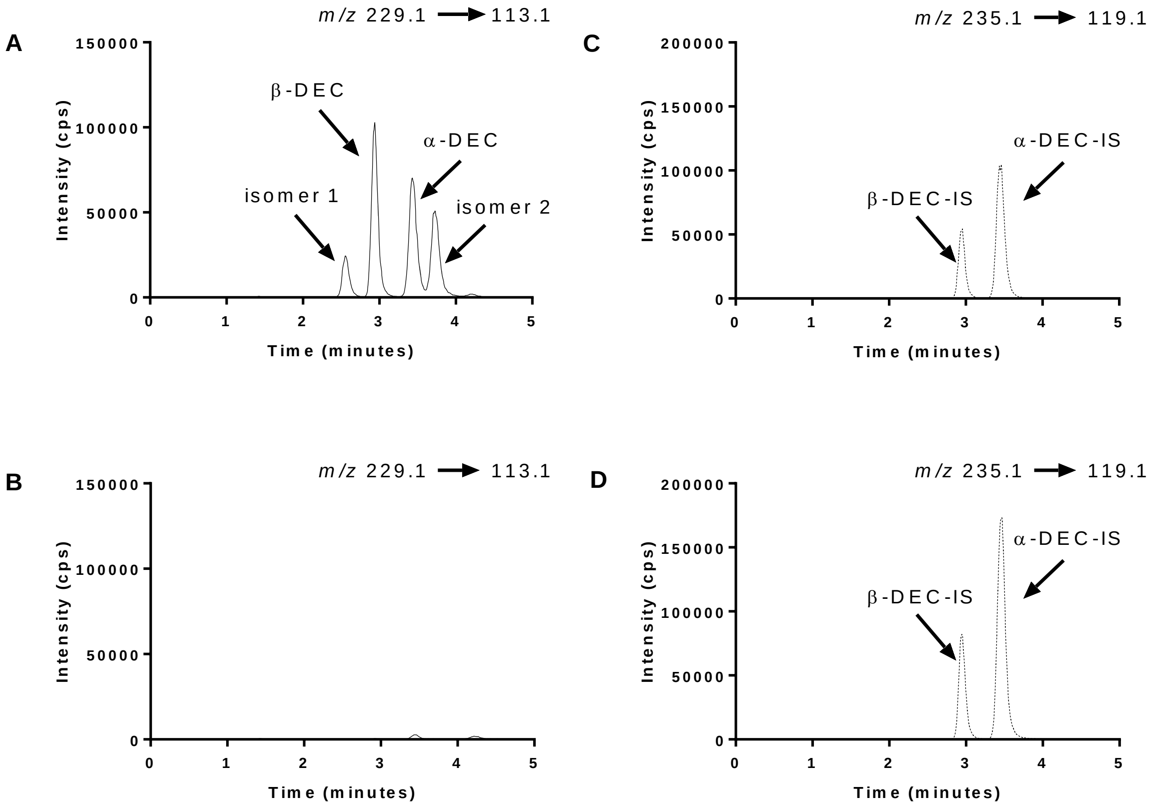

Supplement: Supplementary file 1 — (PDF 539 kb) [file 10637_2019_854_MOESM1_ESM.pdf]
